# Supplementary material for: Intravital imaging-based analysis tools for vessel identification and assessment of concurrent dynamic vascular events
Source: Nat Commun. 2018 Jul 16;9:2746. doi: 10.1038/s41467-018-04929-8 (PMC6048163; doi:10.1038/s41467-018-04929-8)
Supplement: Supplementary file 1 — Supplementary Information [file 41467_2018_4929_MOESM1_ESM.pdf]

Supplementary information to

Intravital imaging-based analysis tools for vessel identification and assessment of concurrent dynamic vascular events

Honkura et al.

Content:

Supplementary Figure 1, pertaining to main Figure 2
Supplementary Figure 2, pertaining to main Figure 3
Supplementary Figure 3, pertaining to main Figure 3
Supplementary Figure 4, pertaining to main Figure 4

Supplementary note 1; Walk-through and trouble-shooting guide,
including Supplementary Figures 5- 10.

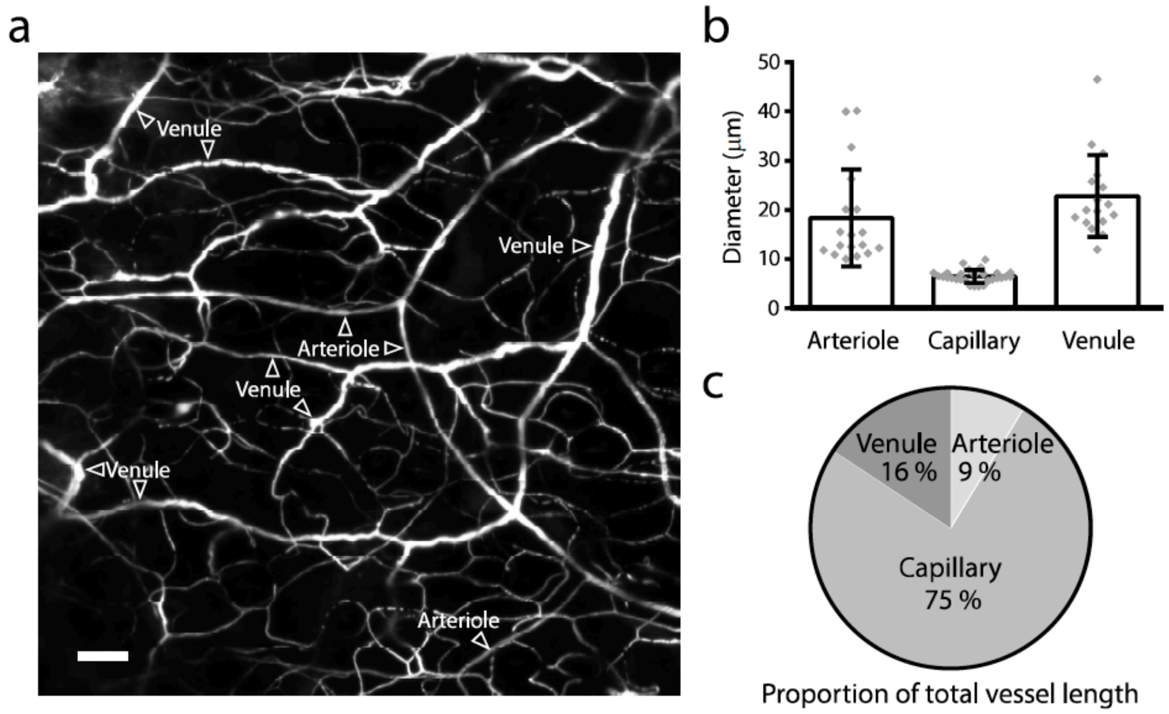

Supplementary Figure 1**. Proportion of vessel types in the ear skin surface**

a. Example of the large field of observation used to measure vessel diameter and proportion of vessel types. Arterioles and venules are indicated. Bar, 100 μm.
b. Quantification of vessel diameter in different vessel types, arterioles, capillaries and venules in the superficial layer of ear dermis. Vessel diameter was measured using Full width half maximum (FWHM; see Methods).
c. Proportion of total vessel length formed by different vessel types, arterioles (9%), capillaries (75%) and venules (16%) in the superficial layer of the ear skin.

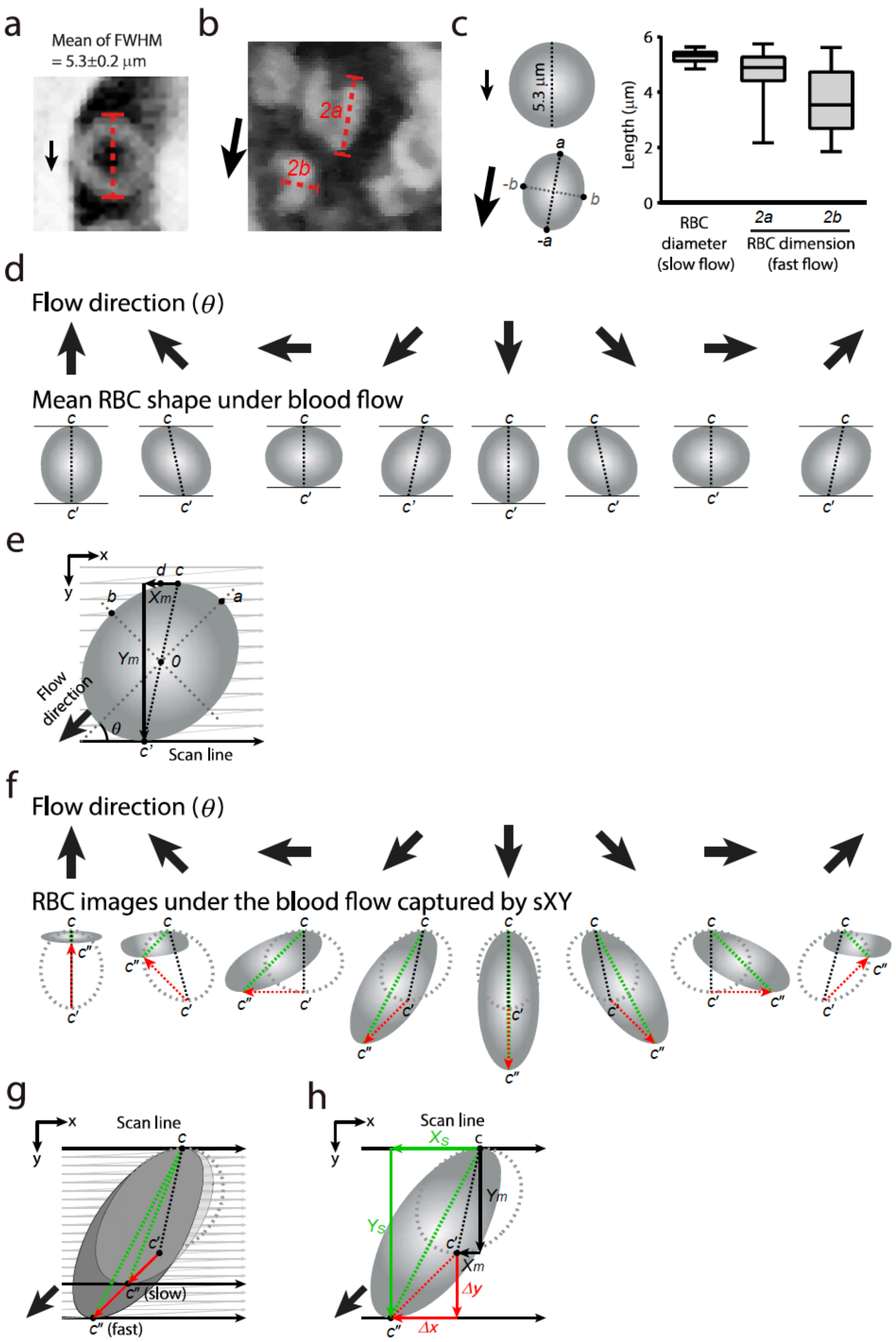

Supplementary Figure 2. **Principles of the RVDM analysis**

a. Typical inverted image of an RBC under very slow blood flow. Mean diameter, 5.3 μm by FWHM. Arrow indicates direction of blood flow. Dashed red line indicates RBC diameter.
b. Typical inverted image of RBCs under flow imaged by fXYT acquisition with parallel (*2a*) and orthogonal (*2b*) axes (red dashed lines) relative to flow direction (black arrow) indicated.
c. Schematic diagram of mean RBC image when imaged by fXYT acquisition under very slow (upper) and normal (lower) flow (left). Co-ordinates *a*, *-a*, *b* and *-b* are the points of contact made with the RBC perimeter by the axes parallel and orthogonal to flow.

Arrows indicate blood flow direction and velocity. Box plot (right) showing RBC dimensions under slow and fast flow following fXYT acquisition with the median (center line), the 25th and 75th percentiles (box bounds) with whiskers (maximum and minimum data points) indicated. n=5 mice with 20 RBCs analyzed for slow flow and 100 RBCs for fast flow (*2a* and *2b*).

d. Schematic diagram of the relationship between flow direction (black arrows) and mean RBC shape, as imaged by fXYT acquisition. Dashed black line in the RBC represents the axis between the points (*c* and *c'*) where the laser scan line forms a tangent with the RBC.
e. Schematic showing co-ordinates (*a*-*d*), flow angle (θ) and erythrocyte lengths (*Xm* and *Ym*) used in RVDM. Co-ordinates *a* and *b* are the points of contact made with the RBC perimeter by the axes parallel and orthogonal to flow, i.e. the longer axis and the orthogonal shorter axis of the ellipse. Co-ordinates *c* and *d* are calculated using equations 1 and 2 (see RBC flow velocity calculation in Methods), where *c* is the point at which the laser scan line forms a tangent with the RBC image and *d* is the point horizontal to *c* and vertical to the RBC center (*0*), used to find lengths *Xm* and *Ym* using equations 3 and 4 (see Methods). Large black arrow indicates flow direction.
f. Schematic representation of distorted RBC shape (solid ellipse) under blood flow captured by sXYT, compared to mean RBC shape imaged by fXYT acquisition (dashed ellipse) under blood flow. Dashed black and green lines represent axes between points where the laser scan line forms a tangent with the RBC for mean and distorted RBCs respectively. Red dashed arrow indicates the difference between lower tangent points (*c'* and *c''*) of RBC images. Solid black arrows indicate flow direction.
g. Representative example of the effect of flow velocity on RBC image shape. Under fast flow (dark grey ellipse; long green line), a larger distance is travelled by the scanning laser before a lower tangent is made with the moving RBC compared to that captured under slower flow (light grey ellipse; short green dashed line), resulting in greater image distortion (compare long and short red arrows).
h. Schematic representation of the method used to find Δx and Δy, i.e. the distance travelled within the laser scan field by a moving RBC along the x- and y-axes respectively. Calculated lengths (*Xm*, *Ym*; black arrows) and measured lengths (*Xs* and *Ys*; green arrows) from distorted RBC images in sXYT respectively, were used to calculate Δx and Δy (red arrows).

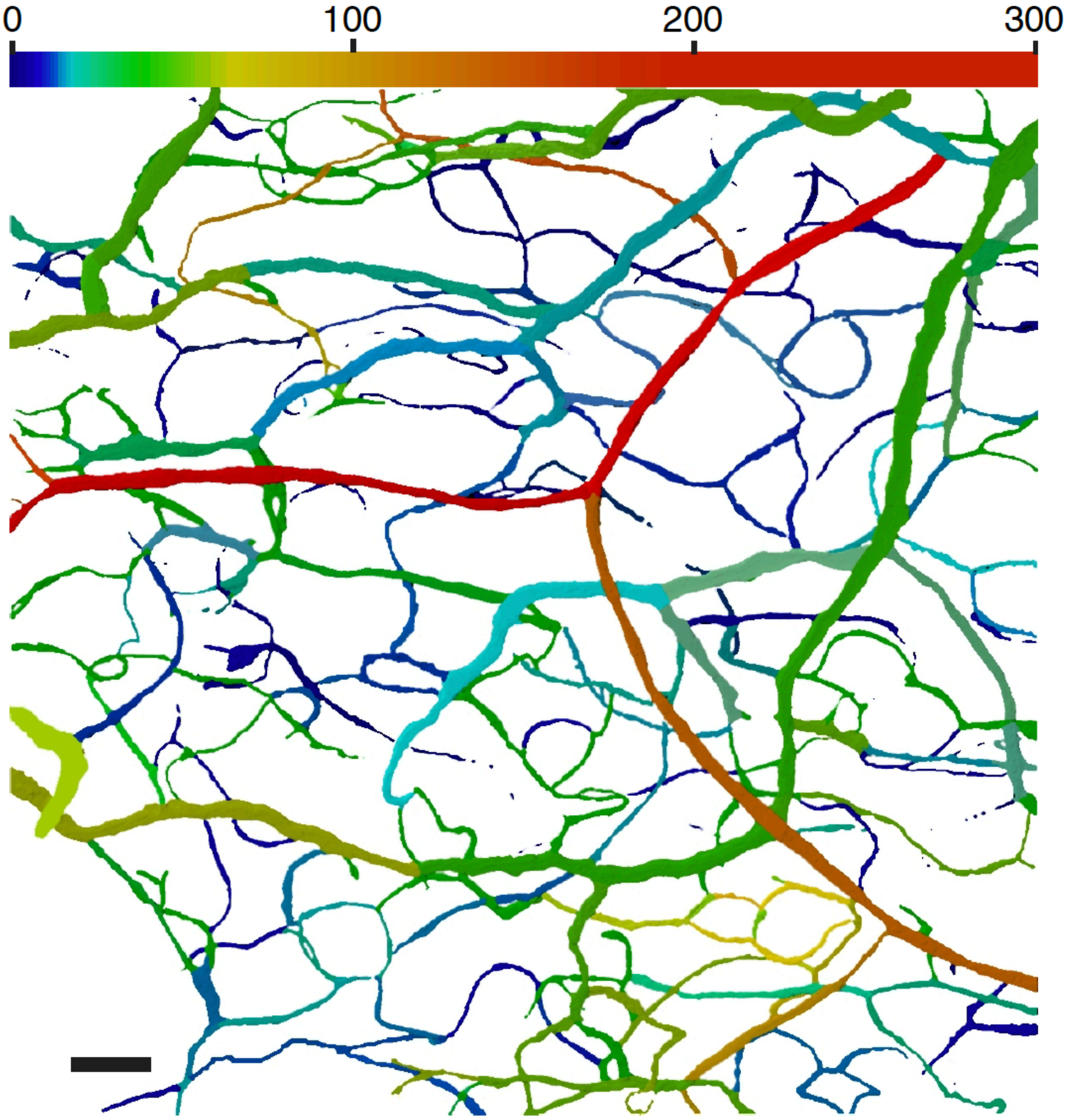

Supplementary Figure 3. **3D velocity map of the ear dermal vascular network**

3D velocity map of the large field of observation shown in Supplementary Figure 1, following RVDM analysis of individual Z-stack images. Color code corresponds to RBC flow velocity (μm/sec). Bar, 100 μm.

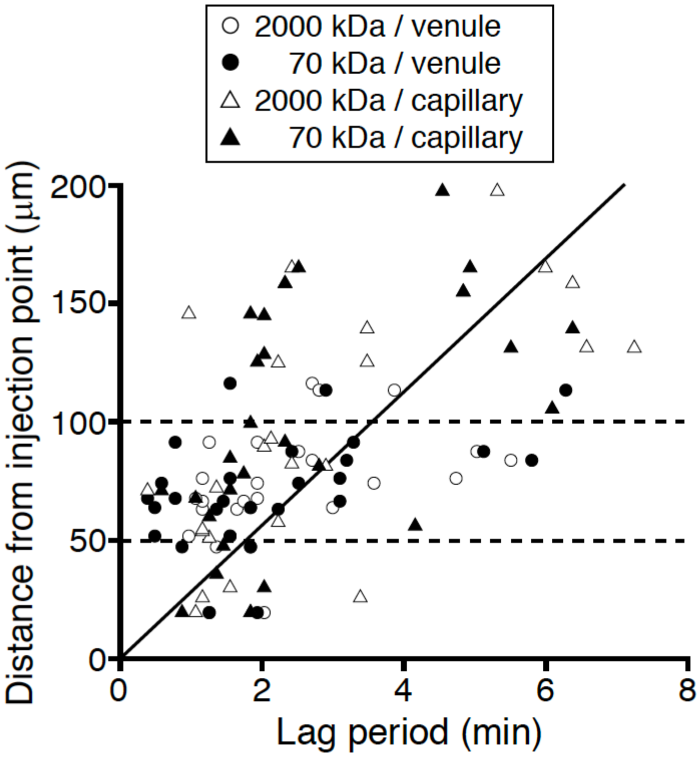

Supplementary Figure 4. **Relationship between lag period and distance from the injection point**

Relationship between the lag period and distance from injection point. Black line indicates regression line. R=0.34.

Supplementary note 1

RVDM method Walk-through and trouble-shooting guide

Calculation of actual RBC size using fXYT imaging

Actual RBC dimensions *2a* and *2b* whilst under flow in C57BL/6J mice are provided in the analysis spreadsheet. Be aware that in certain situations, RBC size and shape may differ from our measured parameters. For example, species or strain variation may exist or may be affected in pathological settings, such as during obese hyperglycaemia [1]. To ensure accurate measurement of flow velocity, it may be necessary to re-calibrate the spreadsheet by measuring actual RBC diameter (*2a* and *2b*) using fXYT imaging. For re-calibration, an acquisition rate under 20 msec / frame is recommended, note however that faster acquisition rates may be required for vessels with very fast flow speeds, to prevent RBC image distortion. From these fast acquisition movies, measurement of RBC length parallel and orthogonal to flow will give *2a* and *2b* respectively (See Supplementary Figure 5). These measurements should be repeated for RBCs under different flow velocities with *n*=30 or higher for accurate measurements. Lengths *2a* and *2b* can then be entered into the spreadsheet in the green highlighted boxes (see Supplementary Figure 6).

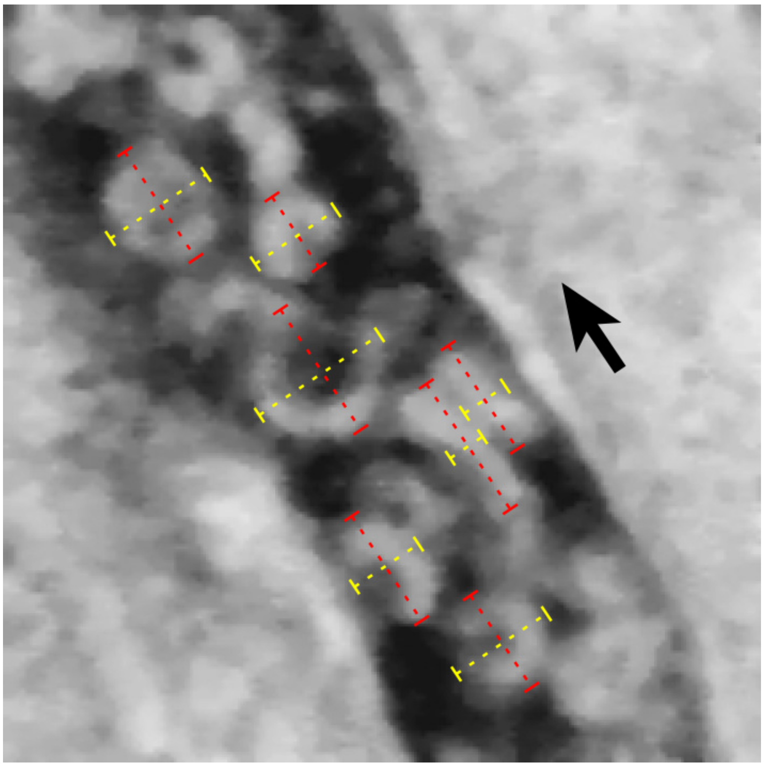

Supplementary Figure 5. Example of an fXYT acquired image used for measurement of actual RBC size. Direction of blood flow is shown by the black arrow whilst dimensions *2a* and *2b* are shown by the red and yellow lines, respectively.

Calculation of flow velocity

Insert the variables from your image highlighted in yellow (see Supplementary Figure 6):

1. Field size (µm)
2. Field size (Pixel)
3. Seconds / frame
4. Flow orientation of the vessel – Measure the angle (0-180°) between the line parallel to scan line direction (See Supplementary Figure 7). NOTE: -1 should be selected when the direction of flow is against the scan direction i.e. from the bottom of the image to the top.
5. Pixel mode – Select µm or pixel depending on the calibration of your image.
6. Analysis type – Select either in-frame or frame.

Erythrocyte dimension 2a	4.8		
Erythrocyte dimension 2b	3.6		
1 - Field size (µm)	200		
2 - Field size (pixels)	1024	0.195	µm/ pixel
3 - sec / frame	1.23	162.34	µm/ sec
4 - Flow orientation	45	1	
5 - Pixel mode	µm		
6 - Choose Inframe or Frame	Inframe analysis		

Supplementary Figure 6. Screenshot of the cells where variables can be inserted in the spreadsheet (highlighted in yellow and green).

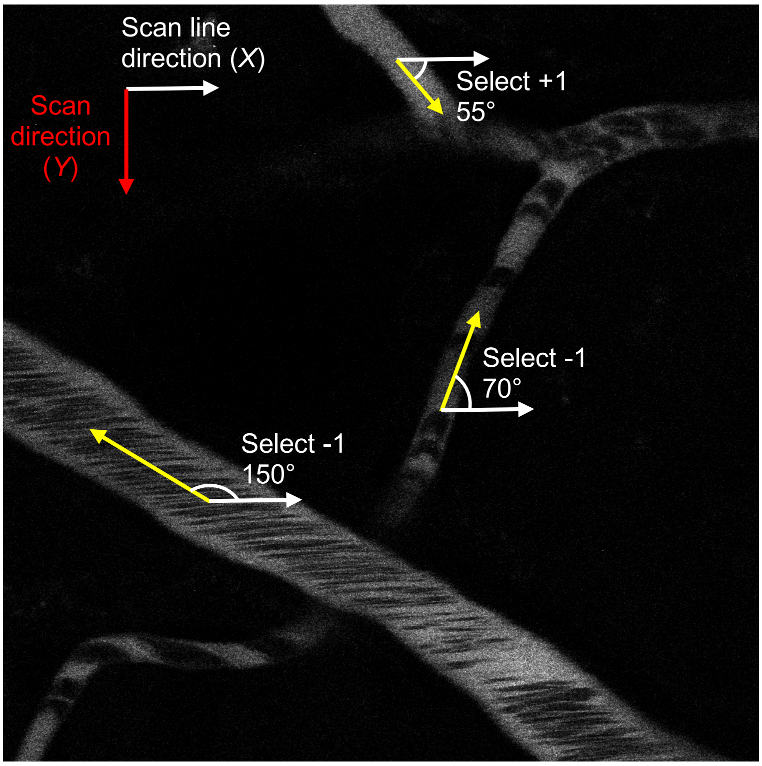

Supplementary Figure 7. Example of how to select flow orientation in relation to scan line direction and scan direction. Note angle size and selection of +1 or -1. Scan line direction is shown with a white arrow, scan direction with a red arrow and flow direction with a yellow arrow.

In-frame analysis

1. Ensure that bounding rectangle is selected to be measured within ImageJ (Fiji) (Analyze → Set Measurements → Bounding rectangle) and that in-frame analysis is selected within the spreadsheet.
2. From acquired movies of flowing RBCs choose an RBC image with a high S/N ratio and clear edges.
3. Draw a line between the upper and lower tangent points formed between the laser scan line and RBC (i.e. *c* → *c'* (not *a* → *a'*) (see Supplementary Figures 2 and 8).
4. Measure the line and insert the line width, height and length into the spreadsheet under their respective headings.
5. Repeat for other RBCs within the same vessel (recommended *n*=20 or higher).

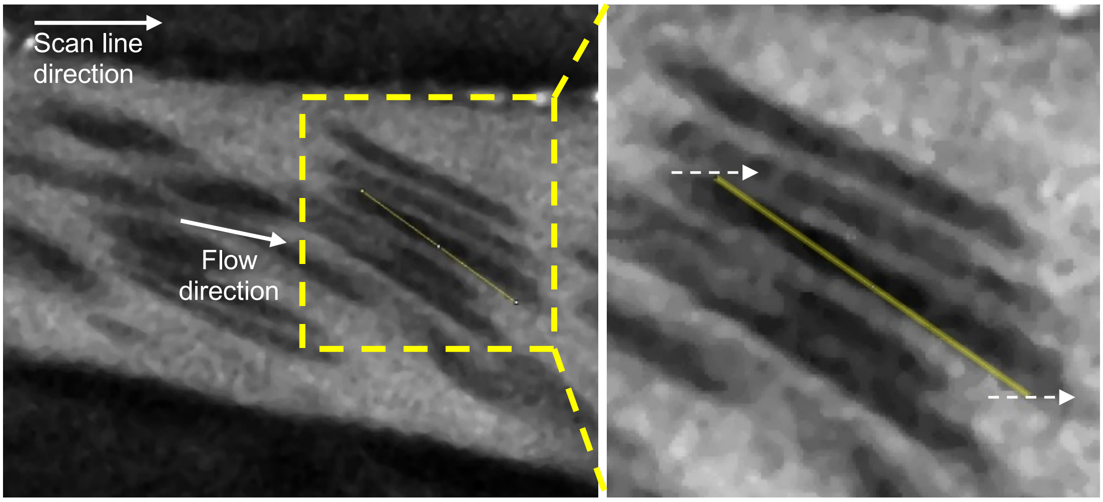

Supplementary Figure 8. Example image of how to measure RBC dimension *c* → *c'* (solid yellow line) between upper and lower tangent points (dashed white arrows).

Frame analysis

1. Ensure that frame analysis is selected on the spreadsheet.
2. Draw a line with ImageJ linking the same point on an RBC between one frame and the next (see Supplementary Figure 9).
3. Measure the line and insert the length into the spreadsheet under their respective headings.
4. Repeat for other RBCs within the same vessel (i.e vessel sections between intersections and bifurcations). Recommended *n*=10 or higher.

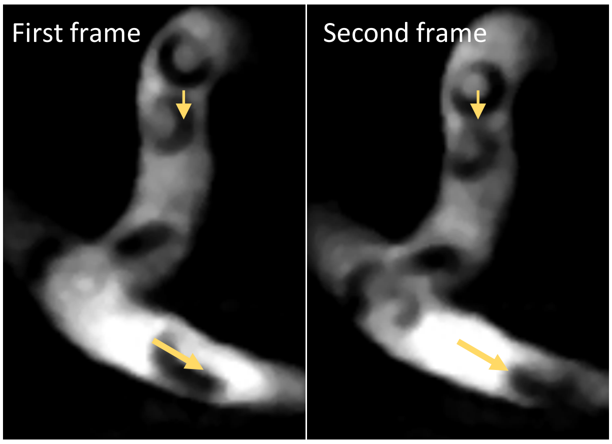

Supplementary Figure 9. Example image of how to measure RBC movement from frame analysis. The yellow arrows illustrate the distance moved by a point on RBCs.

Results

The spreadsheet will calculate the velocity of RBCs from the entered information. The spreadsheet will also determine the reliability of individual data points and reject some based on comparison between *Vx* and *Vy*, the flow angle and their deviation from the mean. The spreadsheet will then return the average RBC velocity, the standard deviation and the number of values that have contributed to these values in the upper left corner.

Troubleshooting

<u>Q: In which direction is the blood flowing?</u>

Determining flow direction is imperative to calculation of velocity but can be difficult. Direction of flow can however be determined from the shape of the RBC image.

For example, if the line *c* → *c'* runs from left to right then then flow is running from left to right and vice versa. See an example in Supplementary Figure 8.

In addition, in cases were flow has a vertical orientation, RBC images will elongate or shrink compared to their actual size when flow is with scan direction (i.e. top to bottom) and against scan direction (i.e. bottom to top), respectively.

<u>Q: How do I know if an RBC image is made of 1 or 2 RBCs?</u>

It is sometimes not clear whether RBC images acquired by sXYT represent single or multi RBC's in vessels with high velocity and high hematocrit.

When measuring RBC dimension *c* → *c'*, single RBC shapes with a clear top and bottom border should be chosen (green arrows, Supplementary Figure 10). The lines indicated by yellow arrows could also be used but might be rejected by the RVDM error checks. The lines indicated by orange arrows shouldn't be chosen for analysis and any measurements taken from these would be rejected by the RVDM error checks.

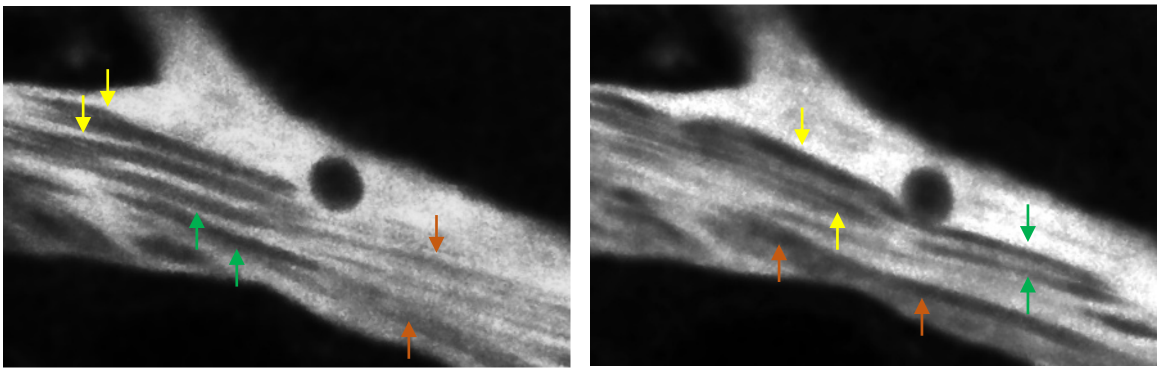

Supplementary Figure 10. Example images illustrating the quality of RBC image, captured by sXYT, which is required for analysis using RVDM. Green arrow, acceptable RBC; yellow arrow, may be tolerated; orange arrow, rejected.

Q: No or very small RBC images appear in vessels; why?
Situations where capillaries have no RBCs running through them can occur when flow is preferentially running through other routes. If this happens then imaging for a longer period may allow for a sufficient number of RBCs to pass. Alternatively, smaller fluorescent particles (no less than 1 μm) may be injected into the blood stream and used to calculate flow velocity.

This situation may also arise when flow velocity is too fast for the scanning parameters, resulting in the laser being able to capture an insufficient portion of the RBC before it passes. In these situations, parameters should be changed to enhance scanning speed.

Q: What do I do if the data I insert into the analysis spreadsheet produces no results?
As the formulas used to calculate velocity reject those data deemed to be unreliable, for example because an RBC image is actually 2 adjacent RBCs or the S/N ratio is poor, situations may arise where the spreadsheet produces no final result or very few n's. This may be more apparent in vessels where the angle of blood flow θ is near 0, 90, 180 or 270° where measurement of Y_s or X_s is less reliable. In such situations, measurement of more RBCs may be necessary or measurements may be taken from a section of the vessel where θ is farther from 0, 90, 180 or 270°. However, in situations where the S/N ratio is too poor new experimental acquisition may be necessary.

Q: How do I decide whether to use the frame or the in-frame method?
This is dependent on the scan speed that is used, however as the benefit of RVDM over for example fXYT is its compatibility with acquisition of wide fields of view using slower scan speeds, the in-frame method would most commonly be used. The frame method may likely only be used in those situations where blood flow is very slow and a single RBC can be tracked from one frame to the next. For example, for the tissue and scan parameters used in this study approximately 85% of vessels required in-frame analysis.

Q: How much time is required to acquire and analyze a field of vessels?
This is dependent on the size of the field, the number of vessels present and the experience of the user. However, acquisition of a field of vessels should take minutes, to ensure capture of a high-resolution 3-dimensional image. The analysis of flow velocity would then be expected to take 2-4 minutes per vessel for an experienced operator.

Q: Do I need to acquire RBC images at different scan angles and speeds?
In order to carry out accurate velocity quantification using RVDM, additional scan angles should not be necessary. On the other hand, additional scan speeds could be useful for vascular beds that have a very broad range of flow speeds. In this case we believe that only two scan speeds would be necessary, one at a normal scan speed as we have used in this study and one faster to allow analysis of very fast flowing arteries.

Supplementary note reference

1 Engstrom, K. G. & Taljedal, I. B. Altered shape and size of red blood cells in obese hyperglycaemic mice. *Acta Physiol Scand* **130**, 535-543, doi:10.1111/j.1748-1716.1987.tb08174.x (1987).
