# Supplementary material for: Intravital imaging-based analysis tools for vessel identification and assessment of concurrent dynamic vascular events
Source: Nat Commun. 2018 Jul 16;9:2746. doi: 10.1038/s41467-018-04929-8 (PMC6048163; doi:10.1038/s41467-018-04929-8)
Supplement: Supplementary file 3 — Description of Additional Supplementary Files [file 41467_2018_4929_MOESM3_ESM.pdf]

Description of Additional Supplementary Files

File Name: Supplementary Movie 1
Description: Vascular leakage dynamics captured by sXYT. Leakage occurred after injection of VEGFA (cyan) through insertion of the glass capillary in the superficial layer of ear dermis. Green; 2000 kDa FITC-Dextran, Red; 70 kDa TRITC-Ficoll, Cyan; VEGFA with Alexa633. Recording duration: 35 min. Recording frame rate: 5 sec/frame. Playback at 30 fps. Data correspond to Figure 1b and 4e-h.

File Name: Supplementary Movie 2
Description: Dye bolus distribution. Progressive distribution of 2000 kDa FITC-Dextran through arteries, arterioles, capillaries, venules and veins in turn after bolus injection. White color indicates 2000 kDa FITC-Dextran. Arrow indicates the time point of injection of the dye into the tail vein; the disappearance of the arrow corresponds to 4 sec in Fig. 2c. Recording duration: 100 sec. Recording frame rate: 1 sec/frame. Playback at 10 fps. Data correspond to Figure 2a-c.

File Name: Supplementary Movie 3
Description: Vessel diameter and RBC velocity dynamics. Injection of VEGFA in the ear dermis induces vascular leakage of 2000 kDa FITC-Dextran (green) and 70 kDa TRITC-Ficoll (red) accompanied by increased vessel diameter and reduced RBC velocity. Recording duration: 40 min. Recording frame rate: 5 sec/frame. Playback at 30 fps. Data correspond to Figure 5a-c.

File Name: Supplementary Software 1
Description: Relative velocity, diameter and morphology (RVDM) software for assessment of red blood cell dynamics in different vessel categories.
